# Multisectoral partnerships to tackle complex health issues at the community level: lessons from a Healthy Communities Approach in rural Alberta, Canada

**DOI:** 10.17269/s41997-022-00653-5

**Published:** 2022-07-07

**Authors:** Kristen Chaisson, Laura Gougeon, Stephanie Patterson, Lisa K. Allen Scott

**Affiliations:** 1 Provincial Population and Public Health, Alberta Health Services, Holy Cross Centre, 2210 2nd Street SW, Calgary, AB T2S 3C3 Canada; 2 Department of Oncology, Cumming School of Medicine, University of Calgary, Calgary, Alberta Canada; 3 Department of Community Health Sciences, Cumming School of Medicine, University of Calgary, Calgary, Alberta Canada

**Keywords:** Healthy Communities Approach, Multisectoral collaboration, Diversity, Community capacity, Rural communities, Approche des Communautés en santé, collaboration multisectorielle, diversité, moyens de proximité, communautés rurales

## Abstract

**Setting:**

Health inequities exist in rural communities across Canada, as rural residents are more likely than their urban counterparts to experience injuries, chronic conditions, obesity, and shorter life expectancy. Cooperative and coordinated action across sectors is required to both understand and address these complex public health issues.

**Intervention:**

The Alberta Healthy Communities Approach (AHCA) is based on the values and core building blocks of the Healthy Communities Approach, a framework centred on building community capacity to support community-led actions on the determinants of health. Adaptations within the AHCA focused on implementation mechanisms with a 5-step process and supporting implementation and assessment tools for multisectoral team building. Local measurement of change was enhanced and focused on community capacity and multisectoral action stages. Between 2016 and 2019, the AHCA was piloted with 15 rural communities across Alberta with population sizes ranging from 403 to 15,051 people.

**Outcomes:**

While communities piloting the AHCA ranged in the level of diversity of their coalition membership and partnerships, members’ reflections demonstrate that intentional engagement with diverse citizens and sectors is pivotal to collaboratively identifying local assets and priorities and mobilizing cross-sectoral action that will sustainably improve supportive environments for cancer and chronic disease prevention.

**Implications:**

Engaging across sectors, building partnerships, and establishing a multisectoral team increase diversity and can catalyze community-led prioritization and actions for asset-based community development. An increase in diversity may lead to increased investment and sustainability at the community level.

## Résumé

**Lieu:**

Il existe des iniquités en santé dans les communautés rurales de tout le Canada, car les résidents ruraux sont plus susceptibles que leurs homologues urbains de connaître des blessures, des affections chroniques, l’obésité et une espérance de vie plus courte. Une action coopérative et coordonnée entre plusieurs secteurs est nécessaire à la fois pour comprendre ces problèmes de santé publique complexes et pour les aborder.

**Intervention:**

L’approche des Communautés en santé de l’Alberta (AHCA) repose sur les valeurs et les composantes de base de l’approche des Communautés en santé, un cadre axé sur le renforcement des moyens de proximité pour soutenir des actions communautaires sur les déterminants de la santé. Les adaptations de l’AHCA ont porté sur les mécanismes de mise en œuvre de l’approche, dont un processus en cinq étapes et une aide à la création d’outils de mise en œuvre et d’évaluation pour la consolidation d’équipes multisectorielles. Les instruments de mesure du changement à l’échelle locale ont été améliorés et recentrés sur les moyens de proximité et les étapes de l’action multisectorielle. Entre 2016 et 2019, l’AHCA a été mise à l’essai dans 15 communautés rurales de l’Alberta comptant entre 403 et 15 051 habitants.

**Résultats:**

Les communautés dans lesquelles l’AHCA a été mise à l’essai présentaient différents degrés de diversité dans les membres et les partenaires de leurs coalitions, mais les réflexions des membres montrent que la mobilisation intentionnelle d’une forte mixité de citoyens et de secteurs est cruciale pour définir de façon concertée les priorités et les atouts locaux et pour mobiliser une action intersectorielle qui instaure durablement des milieux favorables à la prévention du cancer et des maladies chroniques.

**Conséquences:**

L’implication de plusieurs secteurs, la création de partenariats et la formation d’une équipe multisectorielle accroissent la diversité et peuvent accélérer la définition des priorités collectives et les actions de développement de proximité fondées sur les atouts. Une augmentation de la diversité peut mener à des investissements et à une durabilité accrus à l’échelle des communautés.

## Introduction

The *Healthy Communities Approach* (HCA) has community capacity building and empowerment as two core values to guide community development activities. These values are founded on five key building blocks: (1) community/citizen engagement; (2) multisectoral collaboration; (3) political commitment; (4) healthy public policy; and (5) assets-based community development, that together lead to community actions on the social determinants of health (Canadian Healthy Communities, 2009). Since its inception in the 1980s, the approach has been applied by non-profits and community groups who spearheaded multisectoral collaboration that brought citizens, community organizations, and politicians in dialogue for community-led prioritization and actions that could support asset-based community development (Canadian Healthy Communities, 2009). The HCA has the potential to increase community capacity and support the development of healthy public policies that create a mechanism for action on the determinants of health at the local or municipal level (Dailey et al., 2016; Hancock, 1993; Jackson et al., 2013).

While multisectoral partnerships for addressing complex health issues are not a new concept, current understanding of the partnership experience, the risks and benefits and the supportive structures and processes is limited, particularly in the context of smaller rural communities (Willis et al., 2016). In the *Alberta Healthy Communities Approach* (AHCA) project, we explored the lessons shared by members of newly formed community multisectoral coalitions regarding enhancing diversity of representation in their activities. For the purposes of this manuscript, diversity is narrowly defined as bringing together varied perspectives and experiences by engaging across sectors, building partnerships, and establishing a multisectoral community coalition team. As members worked through the AHCA process, they experienced sector diversity in their own work and informally asking questions such as “who is not at the table?”. Based on those learnings, we proposed recommendations to community organizations and system actors that want to increase diversity of sector-related views and perspectives in collaborative community action for health promotion.

## Intervention

### Setting and population

Between 2016 and 2019, the Alberta Cancer Prevention Legacy Fund (ACPLF)^1^ piloted the AHCA with 15 rural communities across Alberta with a population size ranging from 403 to 15,051 people (Statistics Canada, 2011).

### Intervention

The goal of the AHCA is to enhance collaboration and partnership within local communities that will support capacity building for locally defined and driven actions that strengthen the social, physical, policy, and economic environments.

#### Core components

The AHCA maintains fidelity with the HCA values and building blocks (Canadian Healthy Communities, 2009). Our rural adaptions were based upon the vast differences in available assets. Beyond the lack of typical services and resources that would be expected in urban centres, rural communities may lack the human skills and talents that can more easily be found in urban centres (grant writers, evaluators, project managers, facilitators, etc.). Less human capital requires more intentional collaboration across sectors to leverage the specialized human talents that exist. In addition, the AHCA focused on actions that target proximal determinants of health associated with cancer and chronic disease prevention (i.e., supportive policy, social, economic, and physical environments that promote physical activity, healthy eating, UVR protection, tobacco, and alcohol cessation) while remaining flexible to community-led action on other social determinants of health.

#### Implementation mechanisms

**Fig. 1:**
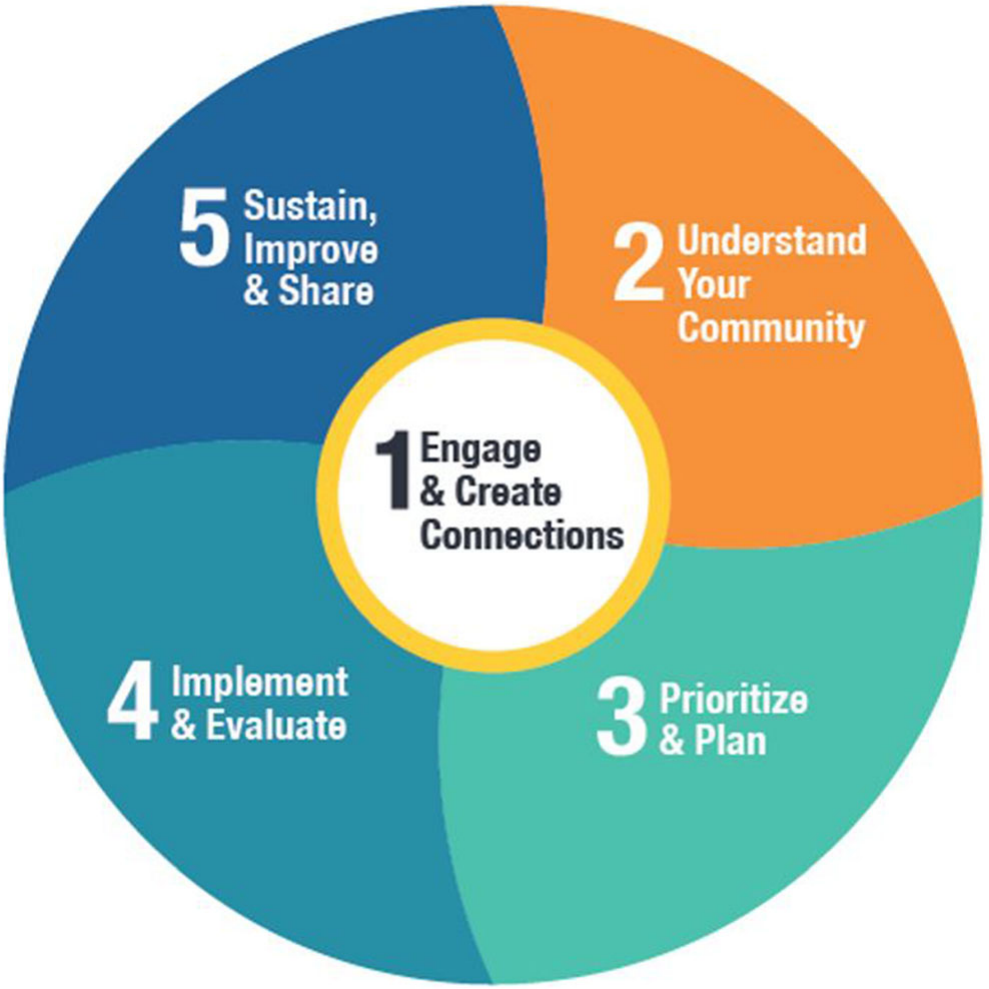
Alberta Healthy Communities Approach 5-step process. The AHCA five-step process is meant to be fluid and flexible (hence its “cycle-like” design). Each step has activities designed to achieve the goal of building the HCA building blocks while ensuring its principles of community empowerment and capacity building. These steps are circular, meaning that coalitions would continuously implement the steps for ongoing actions and long-lasting impact in their community

**Fig. 2:**
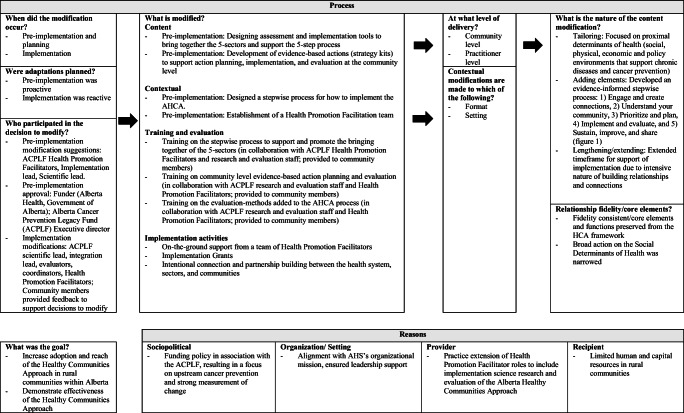
AHCA implementation process adaptations. This figure illustrates how an implementation process was created (producing the AHCA) from the HCA based upon the local rural context in Alberta. Adaptations of the HCA focused on the content (proximal determinants of health associated with cancer and chronic disease) and a structured process (multisectoral collaboration, assessments across sectors, collaborative action, and evaluation planning). The process outlined below follows Stirman et al. (2019) framework for reporting evidence-based intervention adaptations and modifications

The AHCA provides structured implementation strategies (Leeman et al., 2017) co-developed with rural communities in Alberta. We used a community development informed 5-step process (see Fig. 1 and Alberta Healthy Communities Hub (albertahealthycommunities.healthiertogether.ca)). With facilitation from a team of Health Promotion Facilitators, multisectoral coalitions were established in each participating community and engaged in co-designing and piloting tools, mobilizing local assets to plan and implement upstream cancer and chronic disease prevention activities, and carrying out project evaluation. Participating communities received a seed grant of CAD $25,000 to be used in the implementation of local actions. The implementation mechanism adaptations of the AHCA are presented in Fig. 2 using the FRAME-IS method (Stirman et al., 2019).

### Comparison

Descriptions of similarities and differences between communities’ experiences as they went through the AHCA process were collected and analyzed.

### Outcomes

The RE-AIM framework was used to evaluate the intervention with effectiveness defined as increased community capacity and supportive environments. Implementation and effectiveness outcomes were measured at the community level for the initial pilot study. No explicit health equity measures as described in the updated RE-AIM extension for sustainability and health equity were included in this pilot study that occurred between 2016 and 2019 (Shelton et al., 2020).

## Methods

### Coalitions’ sector representation tracking

From 2016 to 2019, tracking of various individuals or groups in the 15 participating communities identified coalition reach to five key sectors in the community.^2^ We used this record to describe the types (counts) of individuals and/or groups to whom the coalition reached out, but the level and the nature of that reach was not recorded. Sector-specific representation was tracked, but no individual identifiers such as age or gender were collected from the coalition members.

### Focus groups

Focus groups were carried out with community coalitions at midpoint (*n*= 7) and endpoint (*n*=11) of the project with all 15 community coalitions contributing to either the mid- or endpoint focus groups. Forty-three community members participated in the midpoint focus groups and 54 participated in the final focus groups. All core members of the multisectoral teams were invited to participate in both focus groups; however, not all members were available to participate. All focus groups were completed in person with an audio recording device, transcribed word for word, and not provided back to the participants.

Focus group transcriptions were coded and analyzed by KC and LG using NVivo 12 software to explore themes related to the coalitions’ reflections on the diversity of their core membership, partnership engagement, collaboration, and hearing and sharing of community voices and stories. The coding instrument was designed by listing key themes that KC and LG expected to be present based on the time-point of the project and focus groups. Next, KC undertook an inter-rater reliability exercise by applying the coding instrument to three midpoint focus groups and three endpoint focus groups. The coding instrument was expanded and continually added to as themes emerged. When a new theme emerged, previously reviewed transcripts were re-reviewed.

A different interview guide was created for the mid- and endpoint focus groups and was not piloted with pilot focus groups. Midpoint focus groups included questions about coalition members’ perceived barriers and enablers of engaging partners and of understanding their community. Endpoint focus group questions touched on the coalition members’ perceived overall experiences of sustainability, and how the AHCA process supported the HCA building blocks and increased community engagement.

Despite the focus groups not including specific questions on diversity or engaging for diverse perspectives, transcripts were coded for insights into members’ perceptions and understandings of diversity across sectors. Quotes shared in the Results section are from the endpoint focus groups; however, both midpoint and endpoint focus groups were thematically coded for theme development. Iterative checking of themes was done through meetings with co-authors (LAS and SP) and through a discussion session with the Health Promotion Facilitators.

## Results

### Coalitions’ sector representation

Eight of the 15 community coalitions reported engagement with groups or individuals representing four sectors, and seven coalitions engaged across all five sectors, mainly during step 2 of the AHCA which focuses on understanding your community. Table 1 presents the different types of sector representation from the 15 communities.

**Table 1:** The total unique types of representation across the 15 communities (calculated by adding together the type of representations for all communities) includes 33 for community facilities and organizations, 16 for community at large, 15 for healthcare facilities, 12 for schools, and seven for workplaces. The top four representations are listed to highlight the diversity per sector

Sector and number of communities that had sector representation	Top four examples of representation types per sector	Number of communities with representatives
Community facilities and organizations (*N*=15)	Recreation facility, society, or coordinators	10
	Community/support services	9
	Library or library society	5
	Parent, family, early childhood groups, society, or centre	5
Community at large (*N*=15)	Volunteers	13
	Community members/residents	8
	Community leaders	4
	Town council members	4
Healthcare facilities (*N*=14)	Health Promotion Facilitators in a provincial healthcare authority (Alberta Health Services)	7
	Primary care staff	5 (3 active; 2 retired)
	Public health nurses	5 (3 active; 2 retired)
	Dietitians	4
Schools (*N*=13)	School health promotion facilitator	2
	School board trustees	2
	School principal	1
	Student representative	1
Workplaces (*N*=11)	Local businesses	8
	Chamber of Commerce	2
	Hospitals	2
	Grocery Store	1

### Thematic analysis

The most commonly identified themes on community coalition sector diversity included the following: (1) the importance of bringing people together, (2) how AHCA tools and activities brought intentionality of diverse sector perspectives, and (3) lessons learned and reflections on gaps in sector representation.

#### Theme 1: The importance of “bringing people together”

Coalition members highlighted that the AHCA catalyzed collaboration by “bringing people together”, which in turn increased the diversity of sector contributions and sharing within the coalition and with local community members and groups. The diversity of sector-specific organizations collaborating within the coalition and those leveraged as partners when implementing actions allowed for different and diverse perspectives and experiences to be shared and considered throughout the AHCA process. Coalitions also perceived that this sector diversity, from businesses, to associations, to council members, was an important asset in their work.

“I think the diversity of the group was really beneficial because it allowed us to take back into our own areas so we can share the information pretty broadly.” Community 3

The ability of “bringing people together” seemed to facilitate connections within the community and expand their ability to engage and/or mobilize more groups naturally:

“It’s challenging because to some degree, partnerships and assets are somewhat related, so if I think about some of the discussions we have had around this table, it is often, you know so many people, so you don’t have to cold call them  to get their support. You have got those relationships where… [the individual] calls whoever he needs to call in [a non-profit organization]. [Another individual] calls whoever she needs to call. It just naturally happens, right? You’ve got these great strong relationships in your community”. Community 13

#### Theme 2: AHCA process brought intentionality

By utilizing and completing AHCA community assessment tools, coalitions engaged in dialogue across diverse sector-specific experiences and perspectives. Coalition members shared that the process of participating in meetings, carrying out community assessments, identifying assets, and seeking out partners, enabled them to work closely together and to bring the various perspectives back to the organizations they represented. These activities enabled *intentional* means to facilitate and support challenging cross-sectoral conversations and provide *evidence* to the discussions instead of “what people think is more important”.

“And to give us some direction because it is evidence. It wasn’t what I think, or this organization, well I think this is more important, right. I think the [supportive environment assessment] tool really helped with that.” Community 6

“I think having that broad representation from community partners… And it is hard work to get people to the table, but I think that was what made it successful.” Community 6

Members identified that the steps of the AHCA process supported their ability to: establish leadership buy-in (organizational, political (municipal leaders), and community champions), strengthen existing connections, develop a shared vision/common goal, and acquire funding (which served as a catalyst for bringing people together and/or as leverage to capitalize more funds and partners to take action). Competing priorities and commitments were a barrier to cross-sector representation in the coalition and made organization and citizen engagement more challenging. It can be suggested that organizational leadership buy-in could be the counter-factor to this limitation, since it would translate into leaders in the organization prioritizing and supporting representation, collaboration, and engagement with the coalition.

“I think the [AHCA] project itself, the benefit of it was getting everybody around the table and I think it quickly became a much bigger vision to take those dollars, add to them and leverage them up and the other groups that we did talk to I think saw that as the benefit of you know... it was one of the first times that they had seen all the groups come to the table and try to use those dollars to create something bigger than what the original vision was. I think that would be the long term benefit is if we can keep all those groups working together.” Community 1

“And in the absence of that [political] commitment, certainly it wouldn’t have happened and that would be a recommendation that I think I would have for future policies that your, policy makers, your town is invested in what you are doing, otherwise” […] “it is a struggle.” Community 9

#### Theme 3: Lessons learned and reflections on gaps in diversity

Some coalition members shared their reflections related to concerns of unintended exclusion, the extent to which each partner organization represented its groups’ interest or their own, the diversity of these groups, the best strategies to engage different organizations and to solidify those relationships for greater citizen engagement, and the challenges of engagement to foster such diversity.

“People should identify early on what group you are representing, for example, why are there three people from one group all voting? […] that was the biggest hurdle to get over or to get established as to… is this really what the community wants or are there two or three people here that are pushing it?” Community 13

Other quotes illustrate the awareness of the limited diversity when engaging the broad community accompanied by the learning that engagement is difficult and needs to be intentional.

“Just talk to all the people in the community. Because you’re not gonna know what they want until you’ve sat down and you’ve actually talked to [community members].” Community 12

“You have to go to them. You cannot host a community information night and expect them to come to you, because they will not. You have to go to them where they are.” Community 12

Engaging certain groups often not included in community activities, such as the youth, requires intentionality and was perceived to have positive impact beyond sharing perspectives, as this other coalition member explains:

“And with the students at the school – because we’re presenting to the Board of Education, the board here – and to the town and city, because student networks are student-led, and so they’re seeing their role in change, and how they can ask for enhanced crosswalks. How they can ask for signage, you know what I mean? And they begin to see themselves not just as children, but as citizens. And know what their roles and responsibilities are moving forward.” Community 12

Coalition members’ insights from focus groups suggest that the opportunities the AHCA created to strengthen local community environments through local collaboration helped to increase awareness of the importance of sector diversity and local mechanisms to increase diversity in their own coalitions. In sum, increasing sector-related diversity in experiences and perspectives in the context of the AHCA was possible through multisectoral partnerships that occur when different groups were brought together to build community capacity and to mobilize for action, as Fig. 1 highlights.

## Discussion

Multisectoral partnerships are essential to forming community coalitions (Butterfoss, 2006; Frieden, 2014) and to building community capacity to address the social determinants of health (Brown et al., 2017). This sectoral diversity in community coalitions and community-led initiatives leads to an increase in communication, collaboration, implementation plans, and sustainability planning (Brown et al., 2017; Hien et al., 2008; Horwitz & Horwitz, 2007), which the communities participating in piloting the AHCA have demonstrated. However, multisectoral approaches can add complexity and difficult power dynamics that may delay community coalition decision-making, consensus building, and planning due to differences in opinions, values, and organizational priorities (Ostrom et al., 1999). Further, relinquishing decision-making power to community groups is a challenge to authorities (political, health), who often have a predefined agenda when engaging with communities (Laverack & Labonte, 2000), leading to potential conflict. Furthermore, priorities and capacity for action in rural communities are influenced by provincial and regional policies, priorities, and funding.

While AHCA communities highlighted that working across sectors is difficult, the benefits outweigh the costs. Similar to Willis et al. (2016), the multisectoral approach brought people and sectors together to create intentional partnerships thus enabling the discussion of “who is not at the table”, thereby advancing equity in local prioritization and decision making. Overall, this work supports the notion that cross-sectoral diversity exists in every community and their interests and priorities are critical to understanding how to influence the complex factors that support health at the community level.

### Limitations

First, the primary purpose of the AHCA pilot evaluation was to examine the effectiveness and implementation mechanisms in rural communities. A diversity-related evaluation question beyond sector representation and ways of working together was not included. However, the local community teams prioritized questions such as “who is not at the table?” beyond the sector representation to understand who was currently being included or excluded. Community coalitions organically had discussions about how to ensure age and gender representation across sectors to increase diversity of individual voices. These discussions are not included in the formal evaluation, and thus are not included within this manuscript. Second, even though tracking of sectors engaged was completed, it did not include the complexity of the collaboration and relationships, such as precise information on coalitions’ core composition and the nature of the engagement with local groups or individuals. On the other hand, the fact that themes of sectoral diversity emerged naturally from other topics may indicate that local leaders in the AHCA coalitions became more aware of how important engagement for diverse sector perspectives is when trying to build a healthy community together.

## Implications for policy and practice

What are the innovations in this program?

- Historically, community health promotion activities have been predominantly top-down with a predefined agenda from public health or government authorities, lacking diverse perspectives from multiple sectors. The AHCA aims to tilt the scale of decision making to the communities, ensuring diverse sectoral representation, with the health authority system serving as a springboard (not the lead) to address local priorities and needs, while levering and building local assets.

What are the burning research questions for this innovation?

- Here we focused on ensuring sectoral representation and diversity as a mechanism to support integrated action on the social determinants of health. Future research needs to focus on multisectoral coalition diversity across age, gender, race, income, geography, and community social capital.
- Both formative research and evaluation should be focused on understanding questions such as the following: (1) are all community populations represented on community coalitions and how can we reach populations not currently engaged; (2) are the impacts of the multisectoral coalitions’ actions experienced equitably across population groups and are there groups more likely to experience negative unintended outcomes; and (3) what are the characteristics of rural settings that do not have the capacity to take on a HCA and how does the AHCA need to be adapted to promote equity across rural settings?

## Data Availability

Not available.

## Footnotes

1 The ACPLF is an interdisciplinary cancer prevention and screening innovation unit housed within Alberta Health Services (AHS), which is the single provincial health authority in Alberta, Canada.

2 Community at large: includes community members who are knowledgeable and/or interested in the community-wide effort that impacts the social and built environment; Community facilities and organizations: Facilities, organizations or groups within the community that provide a broad range of human services and access to facilities; Healthcare facilities: Places people go to receive preventive care or treatment, or emergency health care services; Workplaces: Includes places of employment; Schools: Places where students, teachers, staff, and parents come together in a setting of learning.
